# The determinants of strategic thinking in preschool children

**DOI:** 10.1371/journal.pone.0195456

**Published:** 2018-05-31

**Authors:** Isabelle Brocas, Juan D. Carrillo

**Affiliations:** 1 Department of Economics, University of Southern California, Los Angeles, CA, United States of America; 2 Center for Economic Policy Research, London, United Kingdom; Universita Cattolica del Sacro Cuore, ITALY

## Abstract

Strategic thinking is an essential component of rational decision-making. However, little is known about its developmental aspects. Here we show that preschoolers can reason strategically in simple individual decisions that require anticipating a limited number of future decisions. This ability is transferred only partially to solve more complex individual decision problems and to efficiently interact with others. This ability is also more developed among older children in the classroom. Results indicate that while preschoolers potentially have the capacity to think strategically, it does not always translate into the ability to behave strategically.

## 1 Introduction

Strategic thinking, or the intrinsic ability to anticipate actions and act accordingly, is a cornerstone of rational decision-making. It shapes behavior both in individual situations and in games of strategy. While strategic decision-making in children has received substantial attention in the literature [1–14], little is known about the age at which children start displaying it. For example, tic-tac-toe is popular among preschoolers. However, it is unclear whether children can anticipate multiple steps or whether they are bound to ‘play as they go’ [3]. Similarly, children are known to display some degree of sophistication in interactions, but behavior in most studied paradigms is likely driven by other-regarding concerns, cultural factors, or by mimicking others’ behavior [4, 6, 8, 10, 15–18]. These confounds may hide the contribution of strategic thinking to behavior.

A few recent studies developed paradigms that measure strategic performance in game theoretical settings while controlling for orthogonal concerns [9, 14]. In these studies, children are asked to make decisions and their performance relates to the ability to select an equilibrium strategy. An equilibrium strategy requires acting optimally given an objective, and taking into account that other players are doing the same. These studies suggest that children are not able to strategize, that is to play at equilibrium, before at least 7 years of age. From experimental game theory literature in adults, it is well known that performance in games varies widely across paradigms. Subjects who are able to solve a game requiring a specific number of steps of reasoning may be unable to solve games of similar nature requiring more steps of reasoning [19–22]. This suggests that strategic thinking is multi-facetted and interacts with difficulty. We hypothesize that such effects may have played a role in previous observations in children.

We conjecture that children around 5 years of age exhibit capabilities to think strategically in game theoretic paradigms. On the one hand, by age 5 children have already become less egocentric [23, 24] and have started acquiring the ability to process the intentions of others through Theory of Mind [25–28]. This ability is essential to realize what an opponent might do in a game of strategy. On the other hand, it is still early to perform recursive thinking. Indeed, existing evidence suggests that children are capable of thinking ahead and act accordingly to correct anticipations by 7 years of age [1, 2, 11].

Our main hypothesis is that preschool children are able to think strategically, but their capacity to translate strategic thinking into the ability to solve a task is intimately related to its difficulty. Complexity can come in many forms but it typically relates to pieces of information that need to be processed in order to make a decision. Other things being equal, a multi-stage decision problem is more complex than a one stage problem, a game with multiple options is more complex than a game with only two options, and a game against a real opponent is more complex than a game of chance. It is therefore plausible that, just like adults, the ability of young children to perform strategic reasoning in existing studies is hidden by an excessive complexity of the task.

To test this hypothesis, we present novel experimental tasks specifically designed for preschoolers that allow us to isolate two different aspects of strategic thinking: the ability to perform *logical reasoning* (**LR**) and the ability to perform *anticipatory reasoning* (**AR**). **LR** refers to the minimum level of sophistication necessary to make logical, though possibly myopic, choices. **AR** is the capacity to anticipate future events and use that information to choose the best current course of action. This ability is a simple form of recursive reasoning. We also vary the complexity of our tasks to analyze behavior of children in individual tasks that require simple anticipatory capabilities (**AR-s**), in individual tasks that require complex anticipatory capabilities (**AR-c**), and in games that require taking the perspective of an opponent (**AR-g**).

For analysis, we also investigate the relationship between the ability to think strategically and performance in two paradigmatic tasks of cognitive development in preschool: an egocentrism task and a conservation task. Our egocentrism task helps capture a simple form of perspective taking that is necessary to think strategically. Our conservation task is used to probe logical thinking in a non-strategic setting. This exercise allows us to control for age-related known cognitive developments and to better assess the degree to which preschoolers can think strategically.

## 2 Design and methods

To test for strategic thinking, we recruited 74 children from six preschool classes at a private elementary school across two sessions, one in June 2015 and the other in June 2016. All methods were administered in accordance with existing guidelines and protocol was reviewed and approved by the Institutional Review Board at the University of Southern California (UP-12-00528). We obtained written and informed consent from parents of all participants and oral assent from participants themselves. Children were between 53 and 66 months old at the time the study was administered. Two children were excluded due to not completing all tasks. The remaining 72 children (36 females and 36 males) participated in three Strategic Thinking tasks designed to disentangle between **LR** and **AR**, and three Control tasks to capture known developmental characteristics of preschoolers.

### Strategic thinking tasks

It was essential to find the right level of difficulty for the Strategic Thinking tasks. They had to be easy enough for children 4-5 years of age to solve them and difficult enough to require some degree of anticipation and forward looking reasoning. We designed three novel Strategic Thinking tasks: a Simple individual choice (*Simple*), a Complex individual choice (*Complex*) and a Game (*Game*), that participants played in this set order. While studies addressing developmental stages of strategic thinking usually consider multiple age-ranges [1–14], our research strategy was different. We instead decided to build several games tailored for preschoolers with varying levels of difficulty. These games were, a priori, too complex to understand for 2-3 years-old children and too simple to execute for 7-8 years-old.

**Fig 1:**
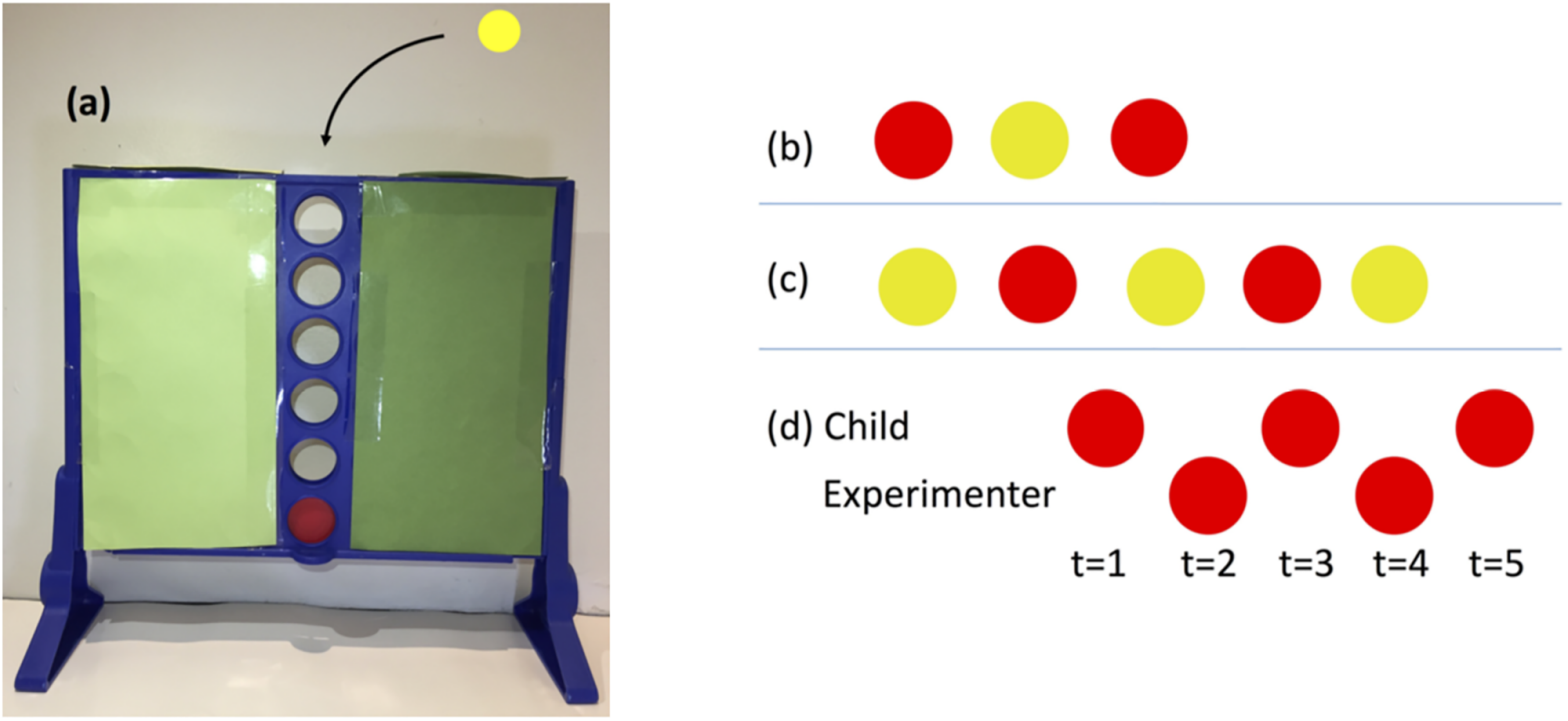
Strategic thinking tasks. (a) One-column “Connect 4” game; (b) In *Simple*, the child must drop tokens in the order Red-Yellow-Red; (c) In *Complex*, the child must drop tokens in the order Yellow-Red-Yellow-Red-Yellow; (d) In *Game*, the child must put exactly one token at each turn.

In *Simple*, the experimenter (she) presented three tokens to the participant (he), two red (R) and one yellow (Y), and instructed him to drop all the tokens in a one-column version of the game “Connect 4” (Fig 1). The participant would win if two tokens of the same color never landed next to each other. Before proceeding, the experimenter asked whether the participant was familiar with “Connect 4” and, independently of the answer, provided a demonstration, emphasizing that tokens would fall in the order they were dropped. In *Complex*, the experimenter added two yellow tokens to the same problem and repeated the instructions. Performing the correct sequences in these tasks (‘R-Y-R’ in *Simple* and ‘Y-R-Y-R-Y’ in *Complex*) required two distinct calculations. First, the participant had to realize that colors must alternate, which called for myopic logical reasoning and therefore captured **LR**. It is a similar ability as the one necessary to solve pattern recognition problems. After dropping a token, the participant could simply look for a token of the other color. Second, the participant had to start with a token of the color that was most prevalent. This, on the other hand, did require to think ahead and captured **AR**. The participant had to anticipate the future consequences of current actions in order to select the correct color. *Simple* required the simple ability **AR-s**, (if I start with ‘Y’, then I will be left with 2 ‘R’, so I should start with ‘R’) while *Complex* required the complex ability **AR-c** (if I start with ‘R’, then I will drop ‘Y’, then I will drop ‘R’, and I will be left with two ‘Y’, so I should start with ‘Y’).

In *Game*, the experimenter allocated two tokens to herself and three to the participant (all of the same color). She explained that both players would alternate dropping as few or as many tokens as they wished, starting with the participant, and that the person who put the last token would win. She also explained that she would try to win. This game is an extremely simplified version of the Hit-N game [29, 30] and the optimal strategy for the experimenter is to always put one token when it is her turn. Therefore, the participant would win only if he also put exactly one token at each turn. This task required **LR** because the participant had to think logically and realize that, in order to keep his advantage, he should never drop more tokens than his rival in the previous turn. This task also required **AR-g**, the ability to anticipate the possible futures after dropping 1, 2 or 3 tokens, which necessitated not only to think ahead but also to take the other player’s perspective.

The two individual choice tasks *Simple* and *Complex* were introduced to the children as problems they needed to solve while *Game* was framed as a game against the experimenter with a set of rules. Even though the children were not acquainted with those problems, they were familiar with the material we used and they were invited to play rather than answer abstract questions. We wanted to avoid proposing conceptual tasks, as it is known that familiarity with context and experience significantly affects children’s performance [31, 32]. At the same time, we wanted to be able to measure children’s intrinsic ability to solve new problems, so we opted for tasks that differed from known games.

Notice that, contrary to standard practices in Psychology, we intentionally decided against counterbalancing the Strategic Thinking tasks to avoid confusing participants. Indeed, there was a natural order to explain the tasks, starting with the simple individual, then moving to the complex individual and finishing with the game against the experimenter. There is a priori no fundamental reason why the order between an individual task and the game should have any effect on behavior, as performance in one did not provide information on how to play the other. Furthermore, tasks were very short and children were given as much time as they needed to relax between tasks to prevent fatigue. By contrast, the order between the two individual tasks could potentially have had a performance effect, since learning to play one of them could provide guidance on how to play the other. We should therefore take this into consideration when interpreting the results.

### Control tasks

Our Control tasks were borrowed and adapted from traditional literature. They consisted of one egocentrism task [23, 24, 33–35] that tested perspective-taking (*Ego*) and two conservation tasks [36] –a non-motivated task with blocks (*Blocks*) and a motivated task with bracelets or bouncy balls (*Toys*). In the egocentrism task, a wall divided two figurines facing each other. We asked each participant if the figurines could see each other. In the conservation tasks, we presented participants with 2 rows: one with 6 objects, and the other with 5 objects. First, we spaced the objects in such a way that the row featuring 5 objects was shorter. We then manipulated the spacing of the objects in front of the child so that the row featuring 5 objects was longer. In the non-motivated version, we asked participants “which line has more blocks?” once before the manipulation and once after. In the motivated task, we asked “which line of toys do you want to bring home and keep?” again before and after the manipulation. The egocentrism task *Ego* captured a very primitive form of Theory of Mind. The conservation tasks *Blocks* and *Toys* captured logical thinking abilities that are known to develop gradually and are not yet fully acquired in preschool. These tasks are useful to study the relationship between logical thinking and the context in which questions are asked. In particular, questions are abstract in the non-motivated version while they are meaningful in the motivated version.

The entire procedure was performed one-on-one between the experimenter and the participant and took 10 to 15 minutes. At the end of each task, children were debriefed on their performance, although the majority realized by themselves whether they had “won” or not. Children who did not complete a Strategic Thinking task successfully could try again if they wished. However, given the endogeneity and heterogeneity in their decision to try again, we report here only the results of their first decision in each task. In addition to the line of bracelets or bouncy balls they decided to keep in *Toys*, children could choose several other toys to bring home (die-cast cars, erasers, trading cards, figurines, poppers, etc.) as a token of our appreciation.

## 3 Results

### 3.1 Aggregate analysis

**Fig 2:**
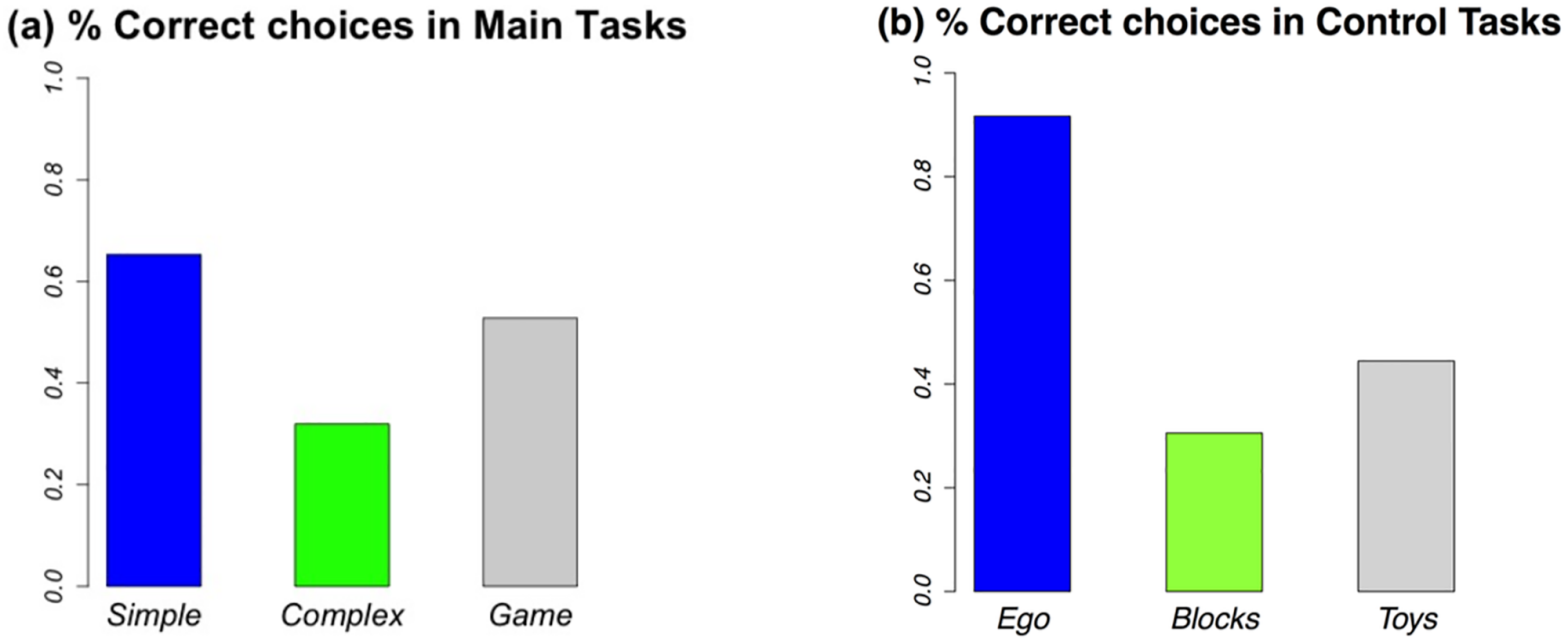
(a) Strategic thinking tasks: Proportions of correct choices in *Simple*, *Complex* and *Game*; (b) Control tasks: Proportions of correct choices in *Ego*, *Blocks* and *Toys*.

The proportions of correct choices in the Strategic Thinking and Control tasks are represented in Fig 2.

#### Individual choice tasks

65.3% of the children solved *Simple* while only 31.9% solved *Complex* and 25.0% solved both. If participants were to choose randomly at each step, they would complete *Simple* with probability $\frac{2}{3}\times \frac{1}{2}=0.33$ and *Complex* with probability $\frac{3}{5}\times \frac{1}{2}\times \frac{2}{3}\times \frac{1}{2}=0.1$, and therefore both tasks with probability 0.03. Our findings are inconsistent with this hypothesis (test for equality of proportions, 95% confidence interval [0.54, 0.75], p-value < 0.001 for *Simple*; 95% confidence interval [0.22, 0.43], p-value < 0.001 for *Complex*; 95% confidence interval [0.16, 0.36], p-value < 0.001 for both tasks).

As an alternative hypothesis, it is plausible that participants understood the need to alternate but did not know which token to choose first. If they were to randomly choose the first token only and alternate thereafter, they would complete *Simple* with probability 0.66 and *Complex* with probability 0.6, and hence both tasks with probability 0.4. While we cannot reject this was the case in *Simple* (test for equality of proportions, 95% confidence interval [0.53, 0.75], p-value = 0.80), participants did not play according to this strategy in *Complex* (test for equality of proportions, 95% confidence interval [0.22, 0.43], p-value < 0.001) nor across tasks (test for equality of proportions, 95% confidence interval [0.16, 0.36], p-value = 0.009). Overall, participants’ performance was better than random and worse than randomly choosing the first token and alternating thereafter.

To better assess the contribution of chance to the overall performance of our participants, we identified children who were able to alternate in both tasks: given any starting color, children were categorized as able to alternate if they chose a different color in the second step and kept alternating until it was not possible to do so (as in sequence ‘R-Y-R-Y-Y’ for instance). We found that 62 children alternated in both tasks. 75.8% of these children completed *Simple* while only 37% completed *Complex*. Children who demonstrated the ability to alternate chose the correct color in the first step more often than random in *Simple* (test for equality of proportions, 95% confidence interval [0.64, 0.85], p-value = 0.01) and less often than random in *Complex* (test for equality of proportions, 95% confidence interval [0.26, 0.50], p-value = 0.0002). Taken together, these results indicate that, on aggregate, while chance may have accounted for some successes, it is unlikely to be a driving force. It is more likely that many children realized that they had to alternate but failed to anticipate with clarity their future moves in order to choose the first token.

Last, it shall be noted that some participants may have transferred knowledge of how to play *Simple* (start with the color most prevalent, then alternate) to their behavior in *Complex*. This means that observed variations in performance may not be entirely driven by changes in difficulty. Given our order of play, the decrease in success between *Simple* and *Complex* constitutes a lower bound of the pure effect of difficulty on choice.

#### Game task

Among our participants, 52.8% successfully completed *Game*. Note that a necessary condition to win was to drop only one token in the first round. If participants were to play randomly in the first round (i.e., dropping 1, 2 or 3 tokens with equal probability), they would win with at most probability 0.33, a hypothesis not supported by the data (test for equality of proportions, 95% confidence interval [0.41, 0.64], p-value < 0.001). Also, among the participants who did not solve *Game*, 20 (58.8%) dropped 3 tokens, and 11 (32.3%) dropped 2 tokens in their first turn. Only 3 children started off correctly by dropping 1 token. This means that children who did not solve *Game* made almost invariably a mistake in the first round.

#### Perspective of others

66 out of 72 children (91.7%) correctly answered *Ego*. This is a higher fraction than in the early literature with preschoolers [23] but comparable to the results obtained in follow-up studies [24]. It indicates very little evidence of egocentrism in our participants. Children were also fast in providing their answers, and could articulate their reasoning (‘they can’t see each other because there is a wall between them’). The overwhelming majority of children were able to take the perspective of others, indicating that failure to complete *Game* was unlikely due to developmental differences in the ability to take a different perspective.

#### Conservation

As in earlier literature [36], more children correctly solved the motivated conservation task than the non-motivated conservation task (44.0% for *Toys* vs. 30.5% for *Blocks*, McNemar test, chi-squared = 4.66, df = 1, p-value = 0.031). Children who answered correctly did so by counting the number of items. None of them applied a conservation reasoning. These results indicate that children in our sample were typical preschoolers.

The main conclusion of this aggregate analysis is that children do not play randomly. They often behave as if they understand the importance of alternating colors in *Simple* and *Complex*. At the same time, they sometimes act as if they fail to anticipate future moves when making current choices in *Complex* and *Game*.

### 3.2 Performance across tasks and classification analysis

**Fig 3:**
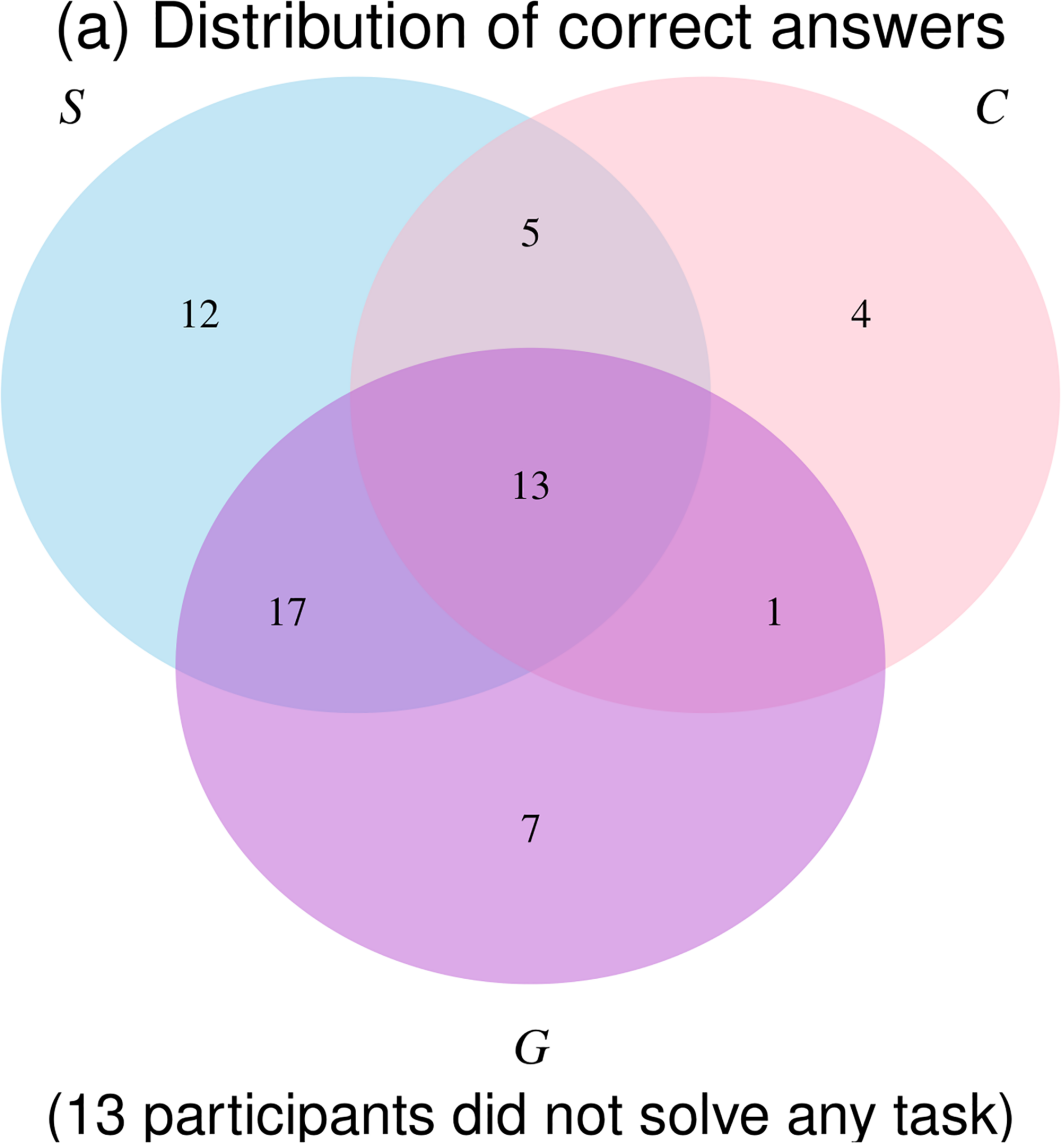
Strategic thinking tasks: Venn diagram showing the number of subjects who solved the different combinations of *Simple (S)*, *Complex (C)* and *Game (G)*.

The previous results suggest that not all children behave equally. We hypothesize here that aggregate results hide developmental differences across children: some children are able to strategize in all tasks while others are still struggling with the most difficult situations. To investigate this possibility and assess heterogeneity in behavior, we represent the distribution of correct choices across Strategic Thinking tasks (Fig 3).

#### Strategic thinking and complexity

27.8% of the children (20) solved neither *Simple* nor *Complex*, 40.3% (29) solved only *Simple* and 25.0% (18) solved both *Simple* and *Complex* (only 6.9% (5) solved *Complex* but not *Simple*). Among the children who solved *Simple* but not *Complex*, 93% started *Complex* with ‘R’ and kept alternating colors until they could not anymore (sequence ‘R-Y-R-Y-Y’). This indicates that these subjects were not playing randomly. On the other hand, among subjects who picked the wrong initial color in *Simple* (sequence ‘Y-R-R’), only 20% completed *Complex* correctly while 65% switched color for the initial token of *Complex* and ended up with the sequence ‘R-Y-R-Y-Y’. These results suggest that many subjects were able to perform the color alternation required by **LR**. Failure was often due to inability to anticipate the correct starting color, or inability to perform **AR**. Table 1 provides a classification of the children according to their logical reasoning and anticipatory reasoning capabilities, after excluding 5 participants who are difficult to categorize due to their success in *Complex* and failure in *Simple*. Children who were able to alternate and solve both *Simple* and *Complex* (they performed **LR**, **AR-s** and **AR-c**) were classified as “strategic thinkers”. Children who were able to alternate but only solved *Simple* (they performed **LR** and **AR-s** only) were classified as “limited strategic thinkers”. Children who were able to alternate but did not solve any task (they performed **LR** only) were classified as “alternators”. Last, children who were not able to alternate in any task (they acted as if they had not yet acquired any of the relevant abilities) were classified as “randomizers”.

**Table 1:** Classification of children according to their behavior in *Simple* and *Complex*.

strategic thinkers	limited strategic thinkers	alternators	randomizers
**LR** & **AR-s** & **AR-c**	**LR** & **AR-s**	**LR**	**none**
18 children	27 children	13 children	9 children

This classification reveals a large heterogeneity, reflecting different developmental stages. The next relevant issue is to investigate how children in different groups performed across our two contexts: individual decisions vs. game.

#### Strategic thinking across contexts

We first investigated whether performance in individual tasks was predictive of performance in *Game*. A Probit regression with the binary variable “success in *Game*” as the dependent variable showed that performance in *Simple* was a significant predictor of performance in *Game* after controlling for demographic variables (Table 2—left). By contrast, performance in *Complex* was not a significant predictor. This result is consistent with the fact that the completion rate of *Game* fell between that of *Simple* and *Complex*, and that these tasks were correctly performed by an overlapping, but non-nested subset of children (Fig 3): *Complex* was perceived as more difficult than *Game* by many.

**Table 2:** Probit regressions of success in *Game*.

	*Game*
*Simple*	0.86 (0.35) *
*Complex*	0.23 (0.34)
*Age*	-0.04 (0.04)
*Gender*	-0.12 (0.31)
*constant*	2.31 (2.71)
AIC	101.41
	*Game*
*Strat*	0.49 (0.38)
**LR** *ability*	-1.30 (0.47) **
*Age*	-0.04 (0.04)
*Gender*	-0.17 (0.32)
*constant*	3.17 (2.79)
AIC	95.91

* = 5% significance (st. error in parenthesis).

** = 1% significance (st. error in parenthesis)

We also found that 72.2% of “strategic thinkers”, 63.0% of “limited strategic thinkers” and 46.2% of “alternators” solved *Game*. By contrast, only 11.1% of “randomizers” solved *Game*. These proportions were significantly different (4-sample test for equality of proportions, χ^2^ = 10.27, df = 3, p-value = 0.016). This suggests that being able to perform **AR** in the individual tasks (especially **AR-c**) transferred to *Game*, though only partially. Moreover, not being able to perform **LR** in the individual tasks was a predictor of not succeeding in *Game*. A probit regression of success in *Game* confirmed that the inability to solve **LR** (dummy variable **LR** *ability*) was a significant predictor of not being able to solve *Game*, while the ability to solve both individual tasks (dummy variable *Strat*) was not, again after controlling for demographic variables (Table 2—right).

These results taken together indicate that the ability to play at equilibrium in *Game* was inherently related to the ability to think logically (**LR**) and to make simple anticipations (**AR-s**) in individual tasks.

#### Strategic thinking and conservation

Interestingly, we found that performance in the motivated conservation task was related to performance in the strategic thinking tasks: 66.7% of “strategic thinkers” were able to solve *Toys* compared to 29.6%, 46.2% and 33.3% of the “limited strategic thinkers”, “alternators” and “randomizers” respectively. A probit regression of success in *Toys* showed that the ability to solve both *Simple* and *Complex* (dummy variable *Strat*) was a significant predictor of solving *Toys*, after controlling for demographic variables (Table 3—left). This suggests that the ability to solve the motivated conservation task is related to the distinctive ability to solve *Complex*.

**Table 3:** Probit regression of success in *Toys* (top) and in *Complex* (bottom).

	*Toys*
*Strat*	0.77 (0.37) *
**LR** *ability*	0.33 (0.40)
*Age*	0.04 (0.05)
*Gender*	-0.35 (0.31)
*constant*	-2.85 (2.70) *
AIC	91.00
	*Complex*
*Toys*	0.71 (0.34) *
*Blocks*	0.13 (0.36)
*Age*	0.08 (0.04)
*Gender*	0.13 (0.33)
*constant*	-5.46 (2.76) *
AIC	91.00

* = 5% significance (st. error in parenthesis).

To assess the relationship between performance in *Complex* and *Toys*, we ran a Probit regression with “success in *Complex*” as the dependent variable. This exercise showed that performance in the motivated conservation task was indeed a significant predictor of performance in the complex individual task after controlling for demographic variables (Table 3—right).

Overall, the ability to solve the arguably most difficult task *Complex* was associated with the ability to rely on intrinsic motivation to overcome the tendency to fail conservation questions.

### 3.3 Strategic thinking and age

The existence of developmental differences in our sample suggested that age, even within our short window, may have played a role in the decisions of participants. We investigated performance in all our tasks as a function of age. We first noted that differences in the conservation tasks were not related to age, again confirming that children in our sample were typical preschoolers.

Children who solved *Simple* were on average older than those who did not (59.5 vs. 57.5 months, two-sample t-test, t = -2.390, df = 50.84, p-value = 0.021). The age-effect was marginal between those who solved *Complex* and those who did not (60 vs. 58.3, two-sample t-test, t = -1.863, df = 38.17, p-value = 0.070). Children who solved both tasks were also significantly older than those who solved neither of them (60.3 vs. 57.2, two-sample t-test, t = 2.788, df = 33.45, p-value = 0.008). In contrast with the individual tasks, we found no significant age difference between children who solved and did not solve *Game* correctly (58.8 vs. 58.9, two-sample t-test, t = 0.120, df = 69.14, p-value = 0.904).

Table 4 reports the average age of children in each category. Age was decreasing in the number of abilities that were correctly implemented. In particular, “strategic thinkers” were significantly older than “alternators” (two-sample t-test, t = 2.183, df = 26.42, p-value = 0.038) and “randomizers” (two-sample t-test, t = 2.959, df = 23.51, p-value = 0.007).

**Table 4:** Average age of children in each category.

strategic thinkers	limited strategic thinkers	alternators	randomizers
**LR** & **AR-s** & **AR-c**	**LR** & **AR-s**	**LR**	**none**
60.3 months	59.1 months	57.4 months	56.9

A multinomial Probit regression confirmed that being classified as “strategic thinker” was positively and significantly associated with age (reference category “randomizers”, coefficient for age in the “Strategic Thinker” category = 0.061, p-value< 0.05) after controlling for gender.

### 3.4 Strategic thinking and gender

We did not find any effect of gender in our study. Female and male solved *Simple*, *Complex* and *Game* at similar rates (all tests for equality of proportions, p-value > 0.05). Within each category, the proportion of females was also similar to the proportion of males (4-sample test for equality of proportions, χ^2^ = 3.493, df = 3, p-value = 0.322). Similarly, performance in the two conservation tasks was very similar (all tests for equality of proportions, p-value > 0.05). These results are also consistent with the regressions reported in Table 2, indicating that performance in the different tasks are correlated but they are not affected by gender.

## 4 Discussion

Our analysis provided evidence that children understood the need to apply logical reasoning, **LR**, in the individual decision-making tasks *Simple* and *Complex*, but many failed to implement anticipatory reasoning, **AR**, in the most difficult tasks *Complex* and *Game*. Our classification analysis revealed a large heterogeneity reflecting different developmental stages: a few participants were able to apply these abilities across contexts while many others struggled with the second, and some did not acquire any. In particular, the ability to play at equilibrium in *Game* was inherently related to the ability to think logically (**LR**) and to make simple anticipations (**AR-s**) in individual decision-making tasks. Interestingly, the ability to solve the arguably most difficult task *Complex* was associated with the ability to answer conservation questions in a motivated context. A few comments are in order regarding these results.

Individual tasks *Simple* and *Complex* were designed to require the ability to perform **LR** and **AR**, but *Complex* was more challenging. In particular, it necessitated more attention to process the information contained in the extra tokens (**AR-c** vs. **AR-s**). The decrease in success between *Simple* and *Complex* together with the significant effect of age on performance suggests that children around 5 years of age are learning to think strategically. The results are consistent with research correlating performance in games with age and difficulty [37] as well as with the well-documented gradual development of executive functions [38, 39]. As executive functions and attention-related mechanisms develop, children learn how to integrate more elements in their decision-making in an efficient way; they progressively learn how to solve simple then complex individual tasks that require anticipation and forward looking behavior. Our results are also consistent with the literature on problem-solving and planning strategies which suggests that young children are able to attend to a limited number of features of the problem. As they grow, they become able to incorporate more information and solve larger problem [40–42]. These ideas are also reminiscent of the fact that young children exhibit centration, the tendency to focus on one salient aspect of a situation, a tendency they overcome only gradually [43].

It was unclear a priori which of *Game* and *Complex* would exhibit a higher success rate. *Game* was cognitively less demanding than *Complex* as it required fewer steps of reasoning (it was relatively easy to discard the alternatives of dropping 2 or 3 tokens). At the same time, it was more challenging because some future moves were left to the discretion of the opponent. While the overall difficulty of *Game*, measured by the likelihood of success, was somewhere between that of *Simple* and *Complex*, the game and individual tasks were correctly performed by an overlapping, but non-nested subset of children. Indeed, among the subjects who did not solve *Simple*, a significant fraction answered *Game* correctly (32.0%). Conversely, among the subjects who successfully solved *Complex*, a significant fraction did not answer *Game* correctly (36.4%). This suggests that the individual and game tasks required related, but different sets of cognitive skills. Interestingly, we also observed that among participants who solved *Game*, 95% realized that colors had to alternate in *Complex*, suggesting that only subjects who were able to perform **LR** were also able to solve *Game*. We also noted that performance in *Game* was predicted by performance in *Simple*. Taking these results together, strategic thinking in *Game* was supported by the ability to perform **LR** and **AR-s**. In turn, this suggests that the inability to select equilibrium strategies in interactive settings may be simply due to limitations of logical reasoning rather than to under-developed Theory of Mind.

Performance in *Complex* was predicted by success in *Toys*. It has been hypothesized that young children, those who are not yet verbally fluent, fail conservation tasks such as *Blocks* because they use a misleading perceptual strategy and answer the question “which line is longer” rather than “which line has more blocks”. This is referred to as the ‘length-equals-number’ heuristic [44, 45]. Recent neuroimaging studies have shown that success in conservation tasks requires the involvement of structures that inhibit the length-equal-number strategy and allow the manipulation of numerical information [46]. These structures are not yet fully developed in preschool children and number conservation is not automatic. It is plausible that motivation facilitates success because it helps children activate attentional processes that are still developing [47, 48]. The fact that success in *Complex* was predicted by success in *Toys* suggests the existence of a common underlying cognitive process that helps both decisions. Given the previous discussion, it is plausible that both complex anticipation and motivated conservation (which is inherently also a complex task for a preschooler) require the involvement of attention-executive processes. In the context of our study, the ability to solve a complex task correlates with the ability to solve a different complex task.

## Supporting information

S1 Dataset (XLS)
